# Supplementary material for: Methoxy-Substituted Tyramine Derivatives Synthesis, Computational Studies and Tyrosinase Inhibitory Kinetics
Source: Molecules. 2021 Apr 23;26(9):2477. doi: 10.3390/molecules26092477 (PMC8122972; doi:10.3390/molecules26092477)
Supplement: Supplementary file 1 [file molecules-26-02477-s001.zip › molecules-1171850-supplementary.pdf]

Spectral data:

Note: All signals coming from solvents residue (ethyl acetate, acetone etc) are not integrated.

Compound Ph1:

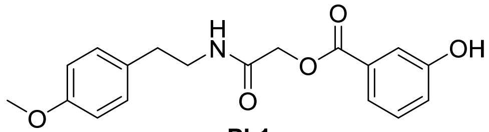

Ph1

1. **^{1}H NMR**

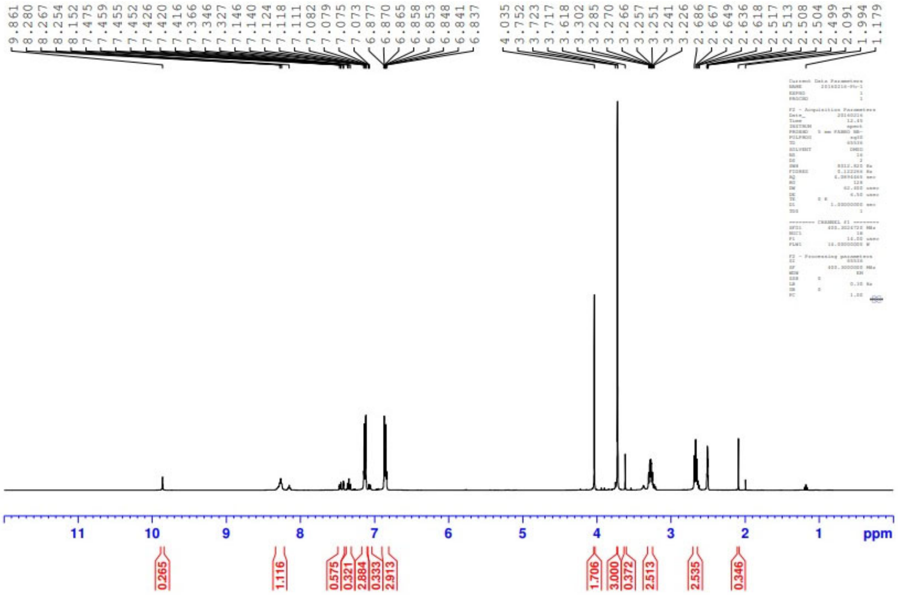

2. **^{13}C NMR**

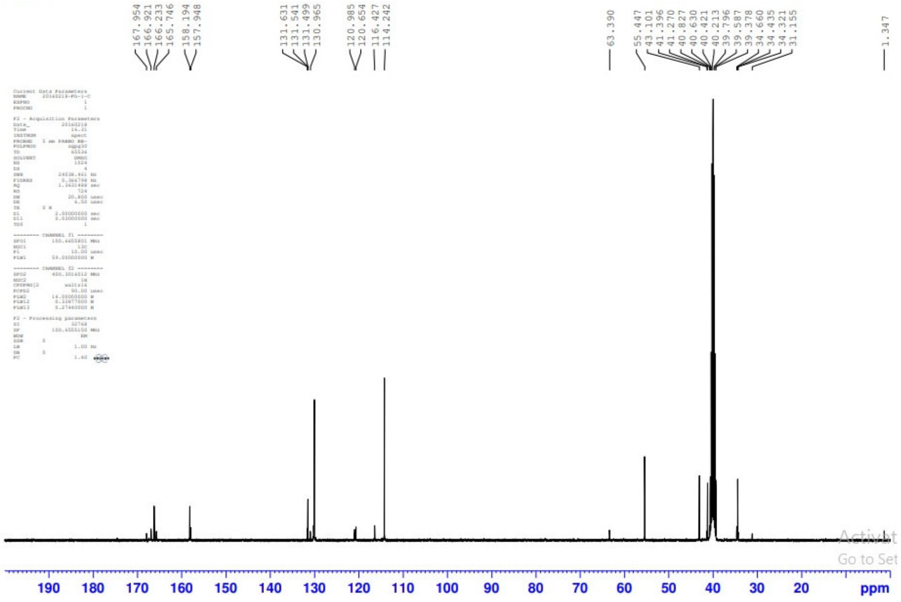

Figure S1. ^{1}H, ^{13}C NMR spectra of compound Ph1

Compound Ph2:

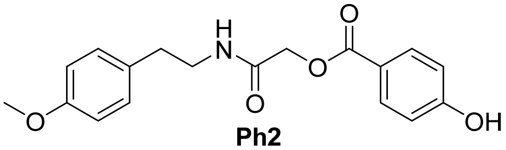

Ph2

1. **^{1}H NMR**

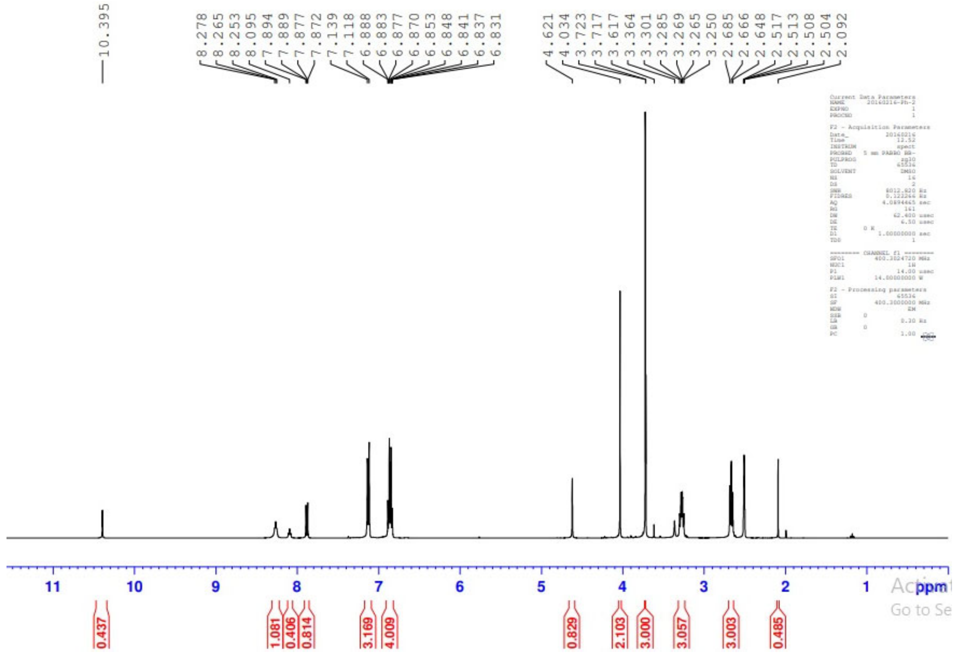

2. **^{13}C NMR**

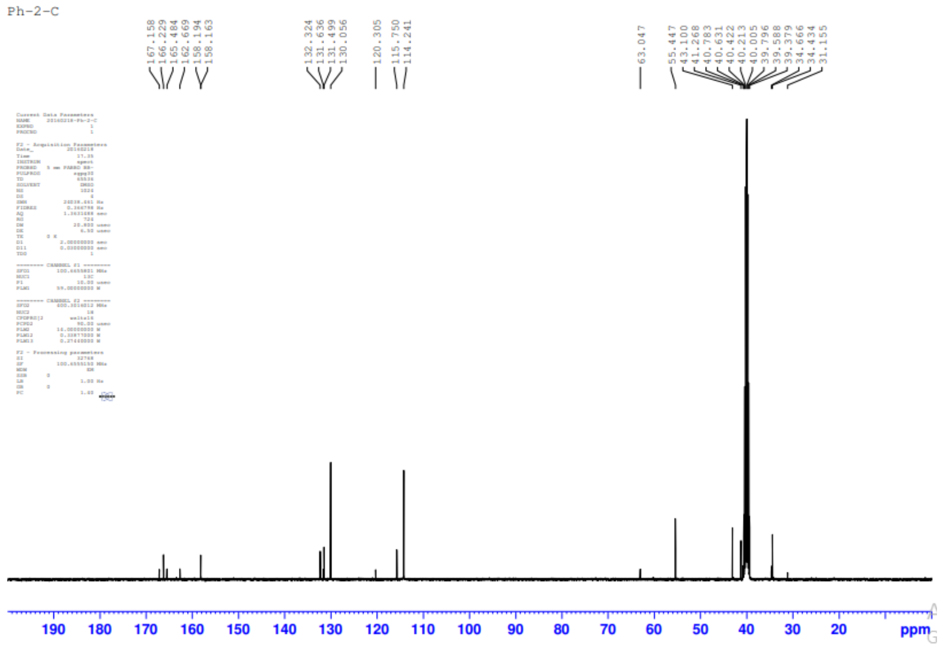

Figure S2. ^{1}H, ^{13}C NMR spectra of compound Ph2

Compound Ph3:

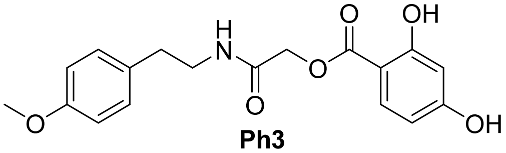

Ph3

1. **^{1}H NMR**

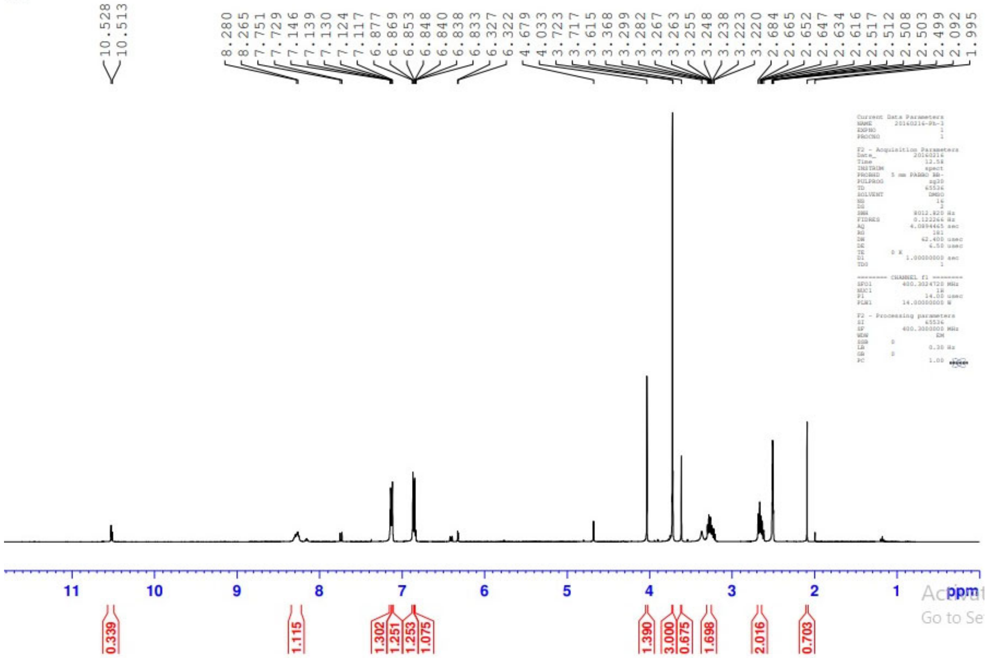

2. **^{13}C NMR**

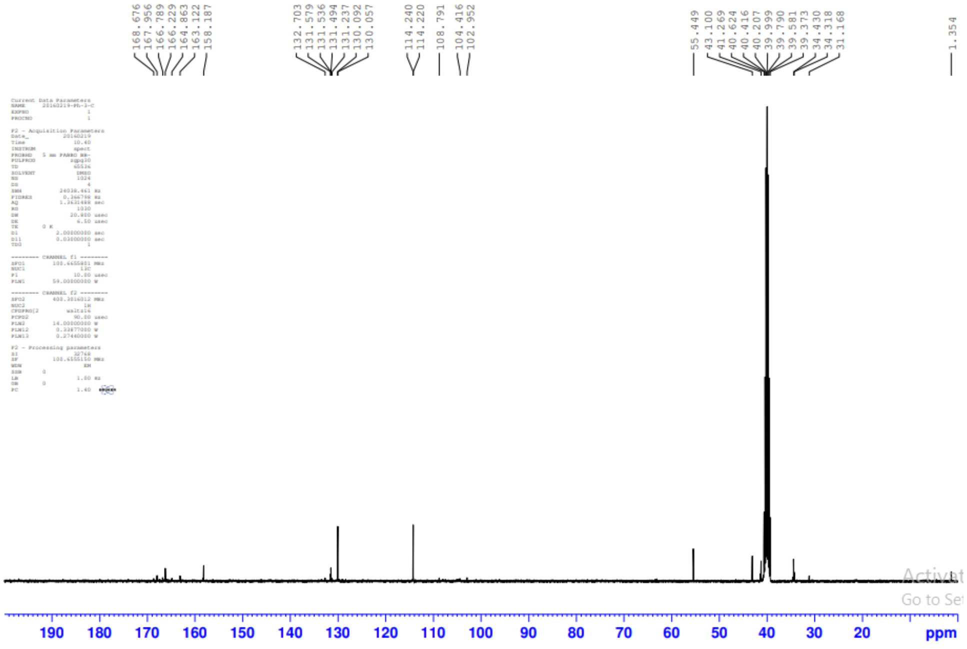

Figure S3. ^{1}H, ^{13}C NMR spectra of compound Ph3

Compound Ph4:

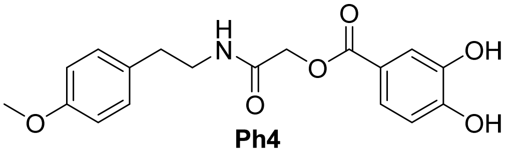

Ph4

1. **^{1}H NMR**

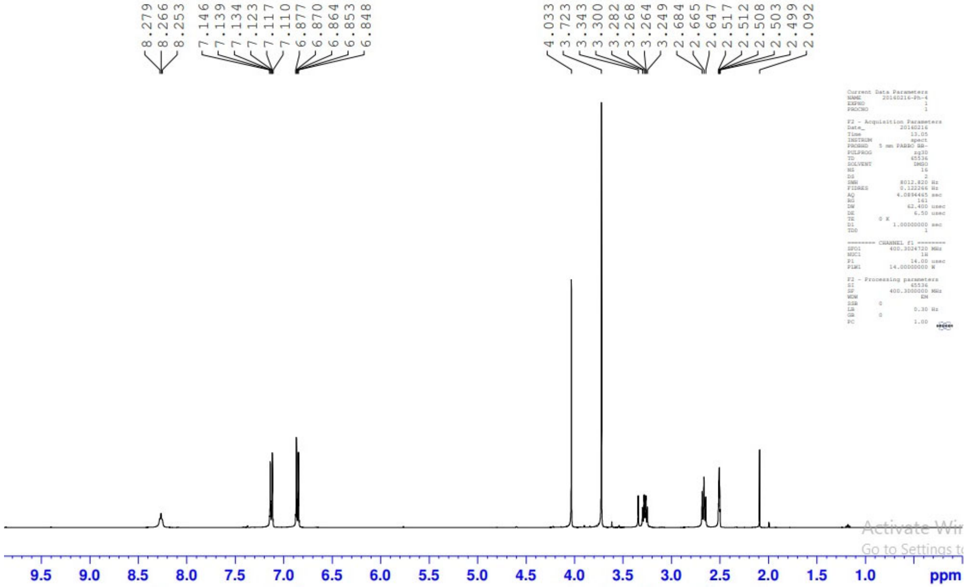

2. **^{13}C NMR**

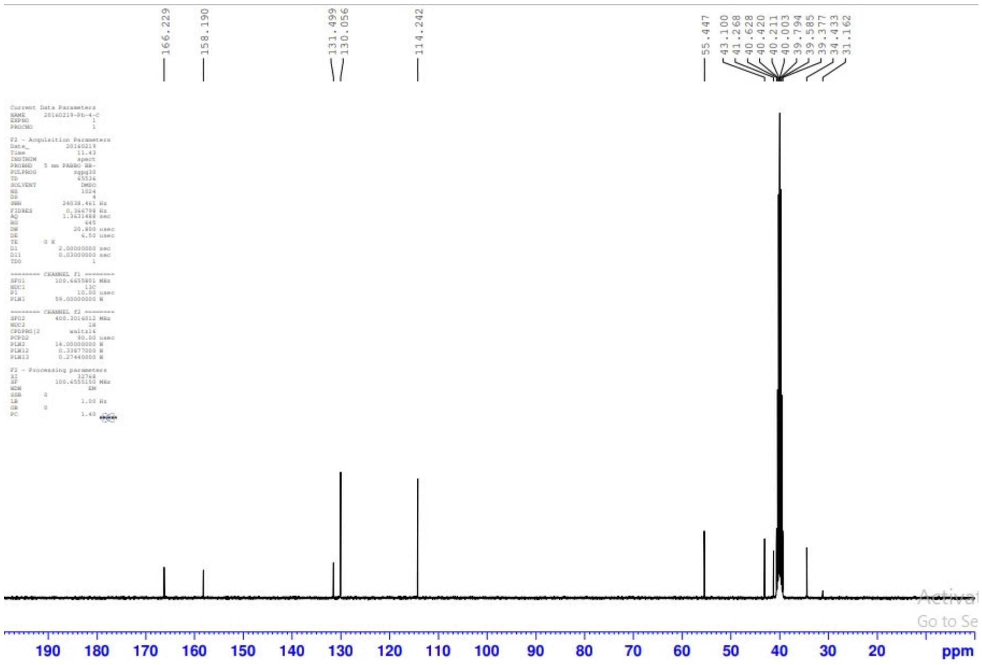

Figure S4. ^{1}H, ^{13}C NMR spectra of compound Ph4

Compound Ph5:

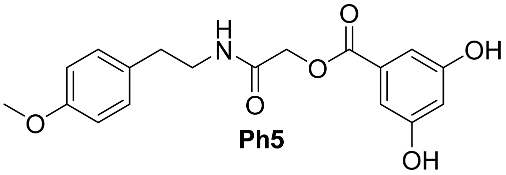

Ph5

1. **^{1}H NMR**

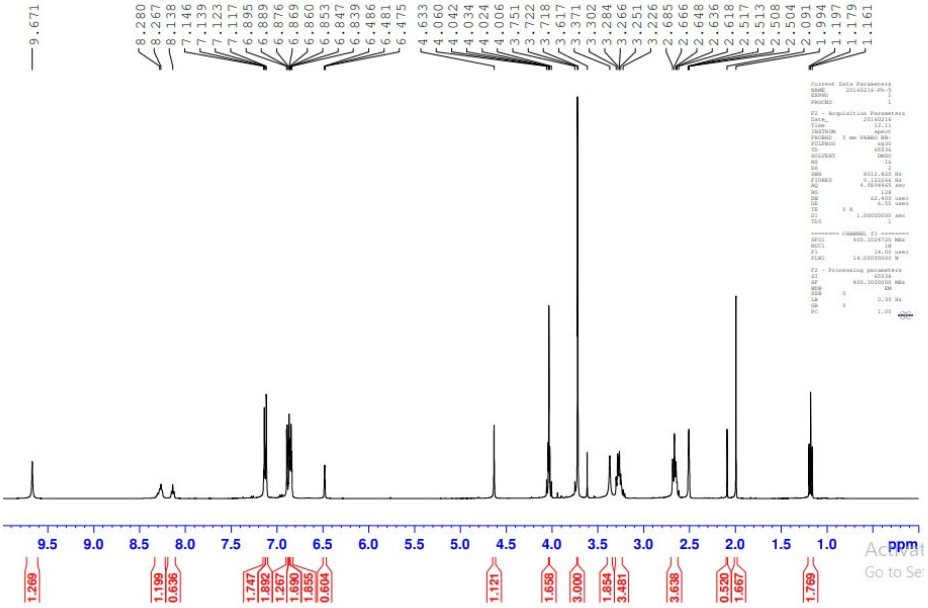

2. **^{13}C NMR**

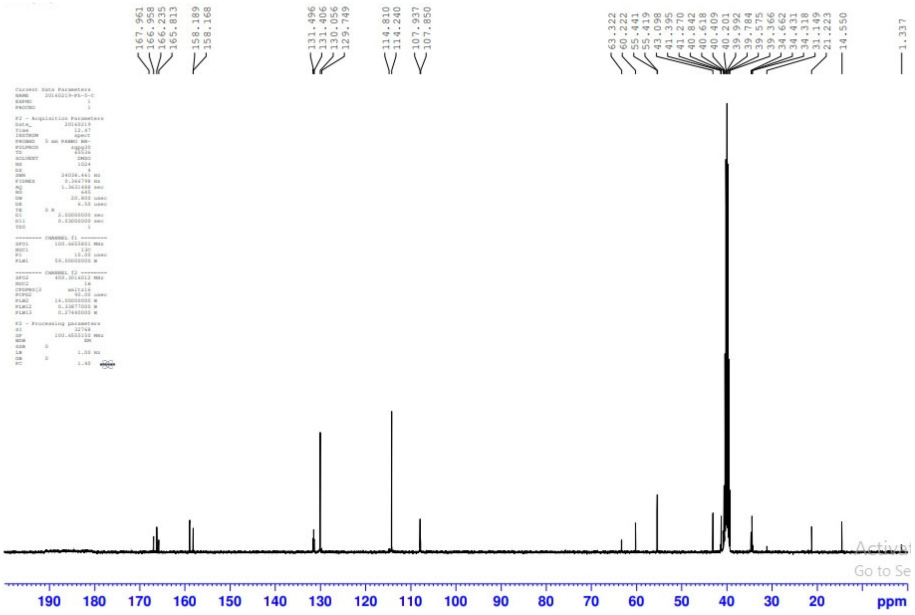

Figure S5. ^{1}H, ^{13}C NMR spectra of compound Ph5

Compound Ph6:

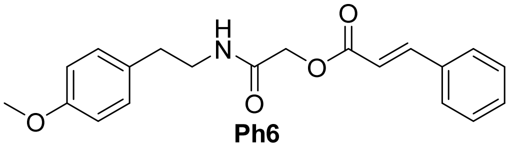

Ph6

1. **^{1}H NMR**

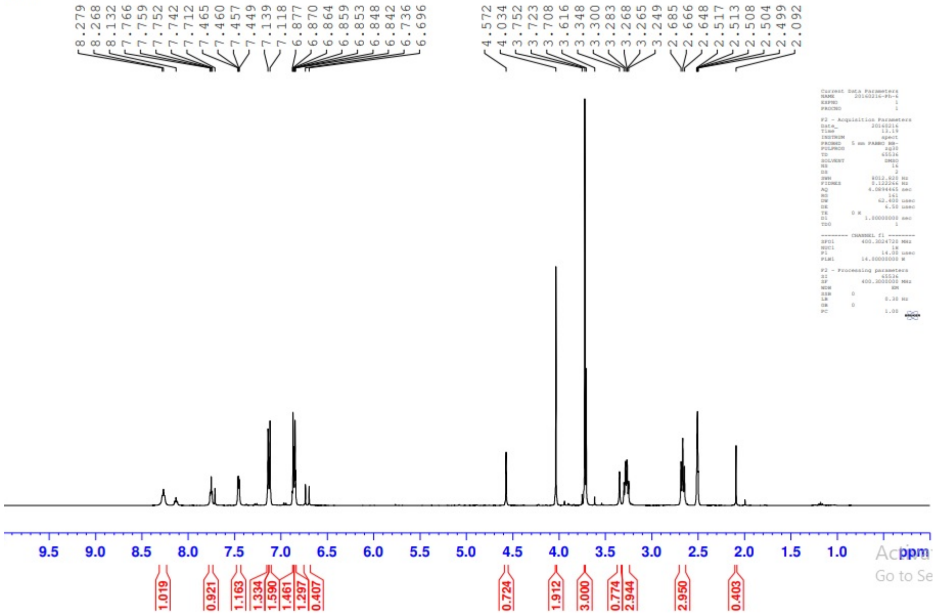

2. **^{13}C NMR**

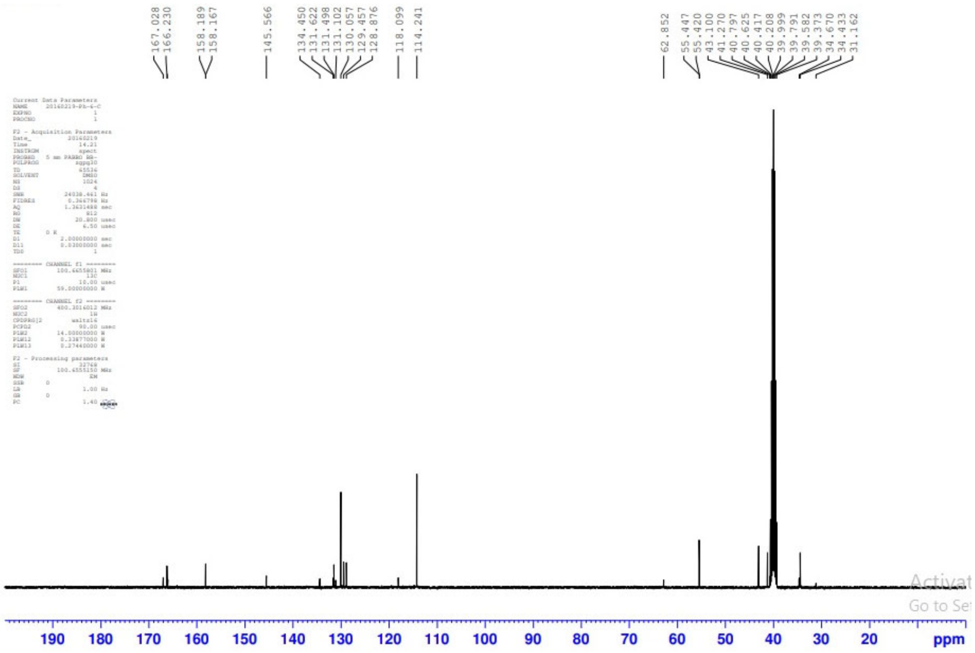

Figure S6. ^{1}H, ^{13}C NMR spectra of compound Ph6

Compound Ph7:

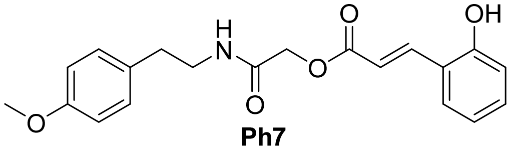

Ph7

1. ^{1}H NMR

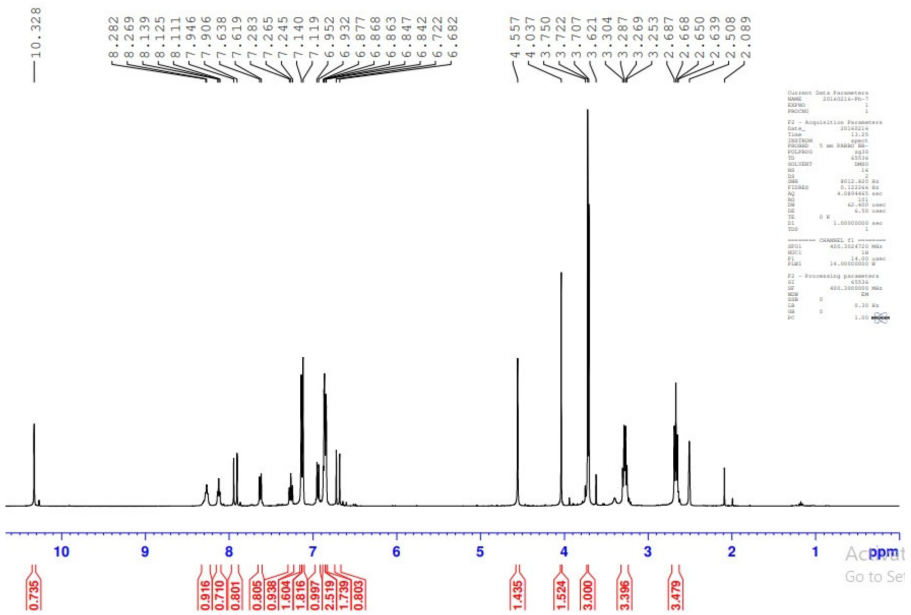

2. ^{13}C NMR

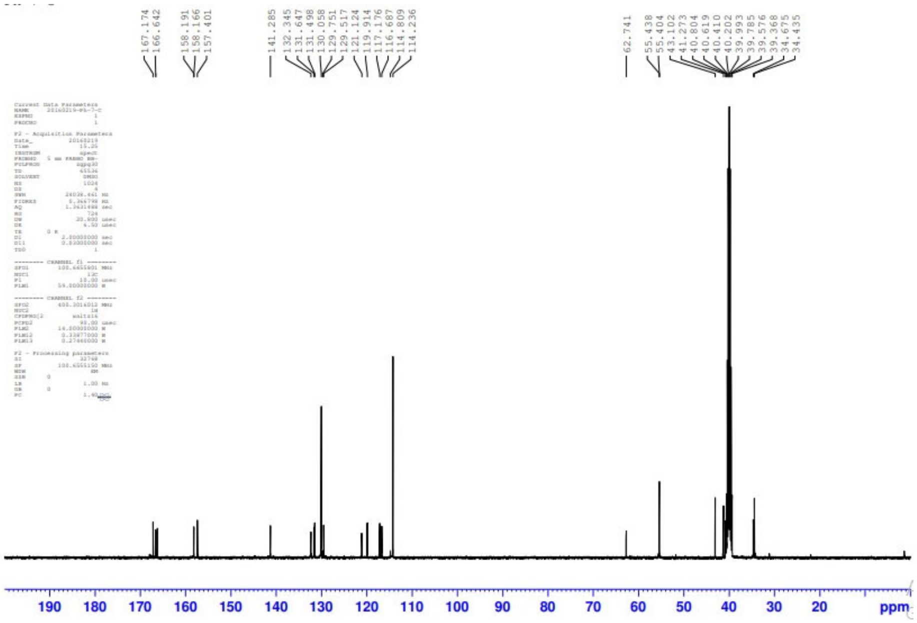

Figure S7. ^{1}H, ^{13}C NMR spectra of compound Ph7

Compound Ph8:

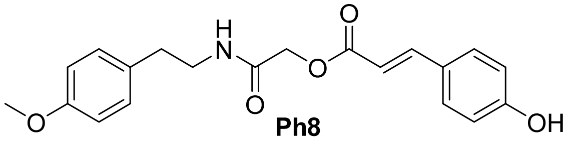

1. ^{1}H NMR

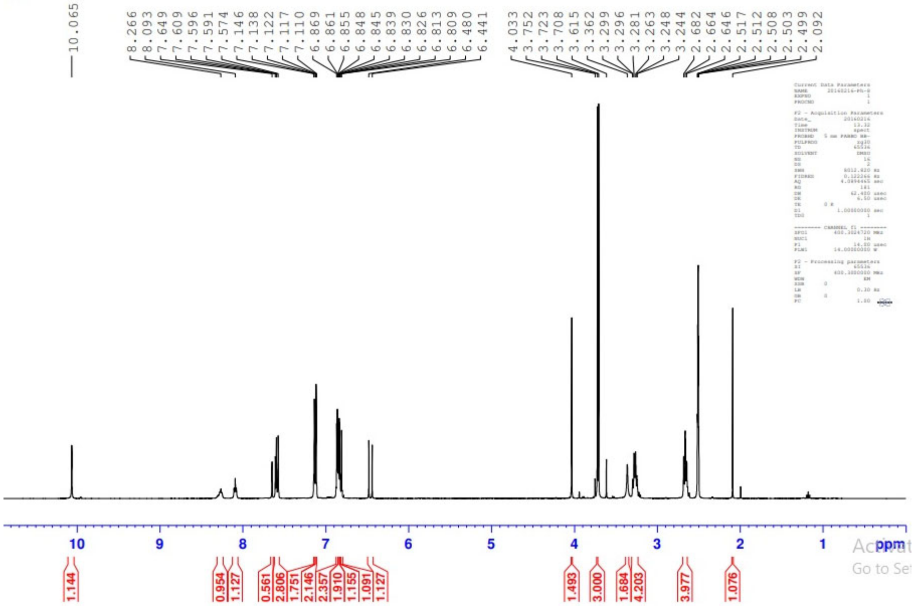

2. ^{13}C NMR

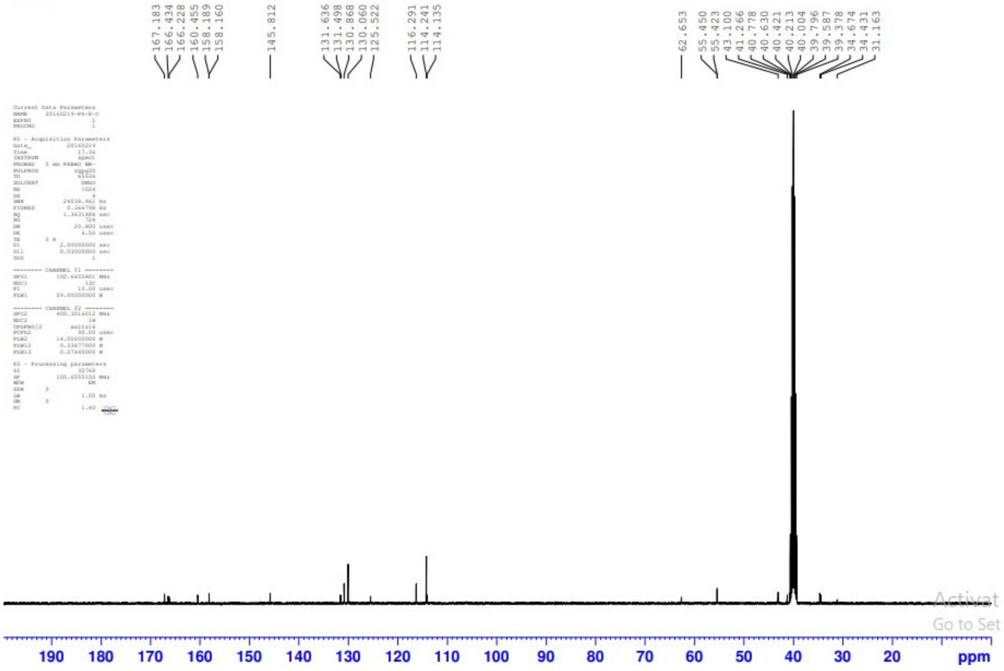

Figure S8. ^{1}H, ^{13}C NMR spectra of compound Ph8

Compound Ph9:

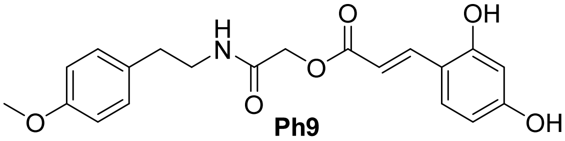

1. **^{1}H NMR**

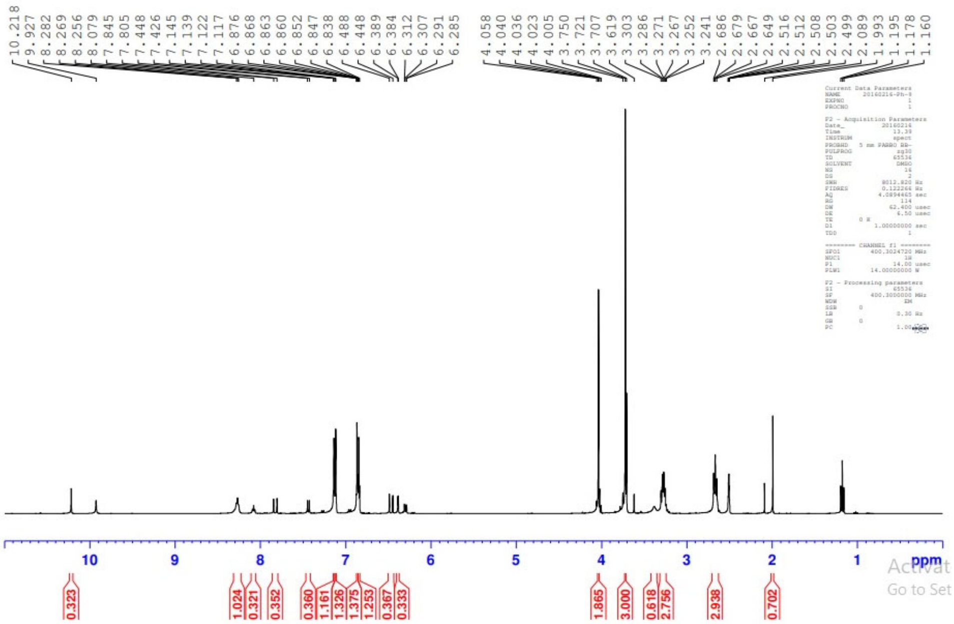

2. **^{13}C NMR**

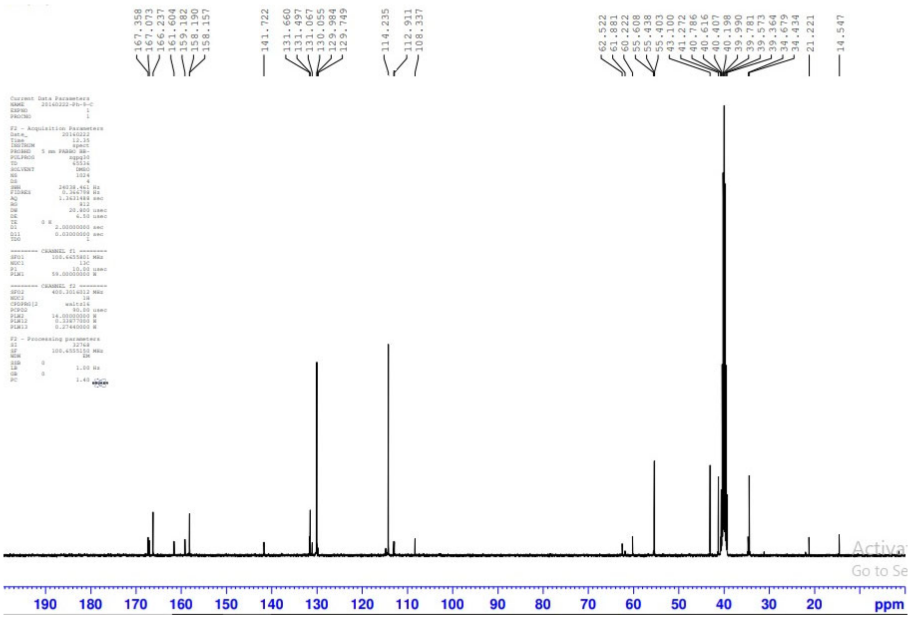

Figure S9. ^{1}H, ^{13}C NMR spectra of compound Ph9

Compound Ph10:

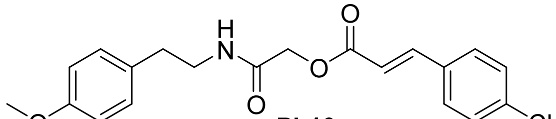

Ph10

1. **^{1}H NMR**

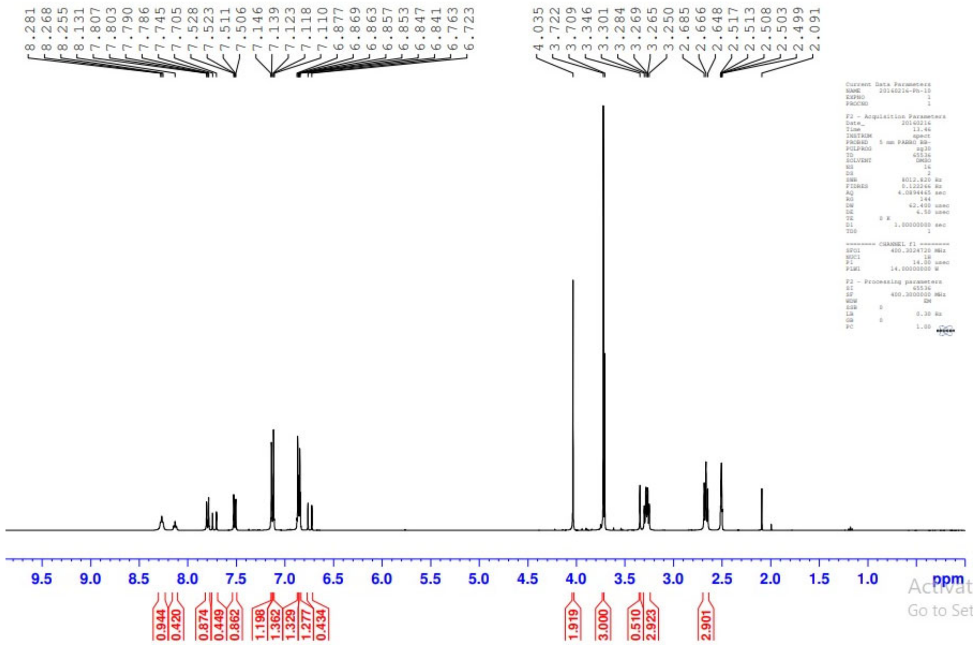

2. **^{13}C NMR**

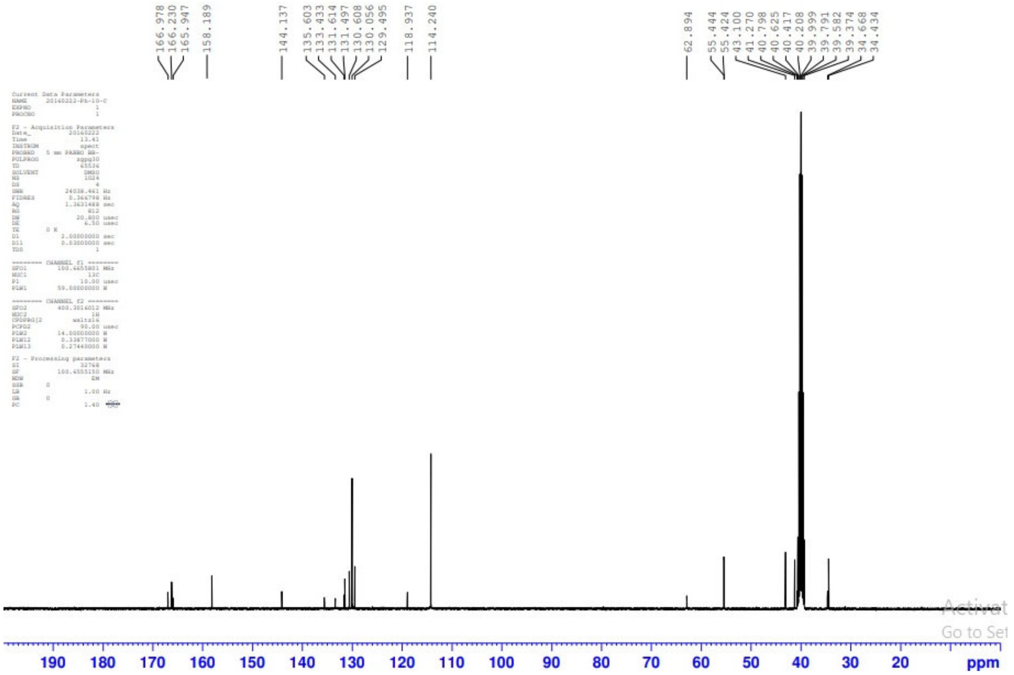

Figure S10. ^{1}H, ^{13}C NMR spectra of compound Ph10
